# Supplementary material for: Multi-dimensional super-resolution imaging enables surface hydrophobicity mapping
Source: Nat Commun. 2016 Dec 8;7:13544. doi: 10.1038/ncomms13544 (PMC5155161; doi:10.1038/ncomms13544)
Supplement: Supplementary Information — Supplementary Figures 1-15 and Supplementary Tables 1-2. [file ncomms13544-s1.pdf]

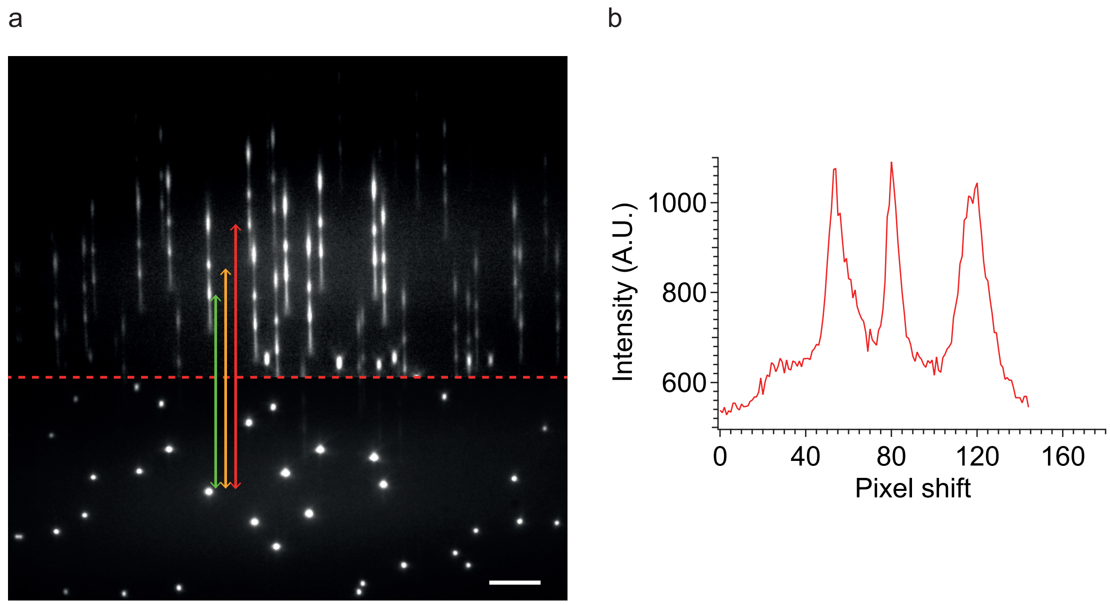

Supplementary Figure 1. Transmission grating spectral calibration. (**a**) TetraSpeck™ beads imaged (contrast adjusted) on the sPAINT instrument. The scale bar is 5 µm. The spatial domain is at the bottom of the image; the beads appear as bright puncta. To the top of the image, the individual spectra from each of the beads are projected. The green, orange and red arrows depict the approximate distances between the spatial position and the three peaks that are required for the analysis (see supporting code, STEP_1_sPAINTCalib.ijm). (**b**) Intensity profile for the spectrum labelled with the three arrows. The three peaks correspond to emission from the green, orange and red fluorophores on the bead.

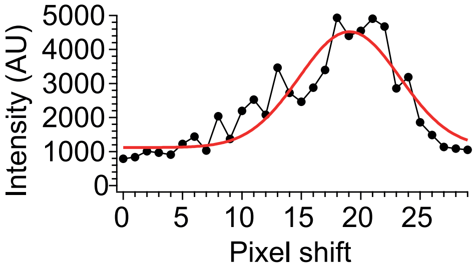

Supplementary Figure 2. Spectrum fitting. Example of a peak extracted (black line) from the spectral part of the image (Tetraspeck^{TM} beads), which was fit to a Gaussian distribution (red line) to determine the centre position.

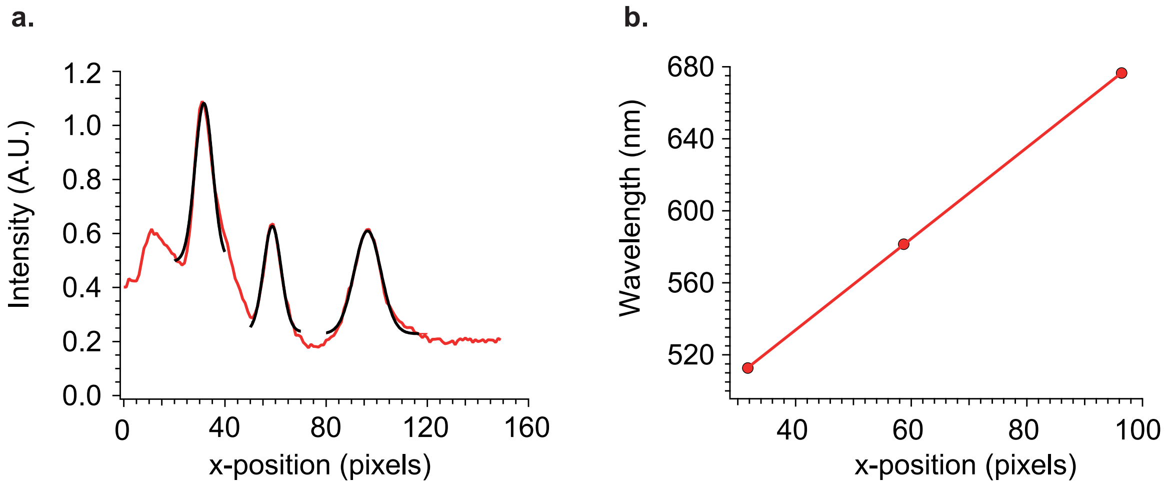

Supplementary Figure 3. Determination of nm/pixel ratio. By exciting TetraSpeck™ beads at 405 nm, three emission peaks are generated (centred at 512.7 nm, 581.5 nm, and 676.5 nm) on the spectral domain area of the chip. The intensities over all localizations are summed (a. red line), and the center positions (in pixels) of the peaks are determined by fitting to three different Gaussian distributions (a. black peaks). These are plotted against their actual wavelength values, and fitted to a straight line (b.) to determine the nm/pixel ratio.

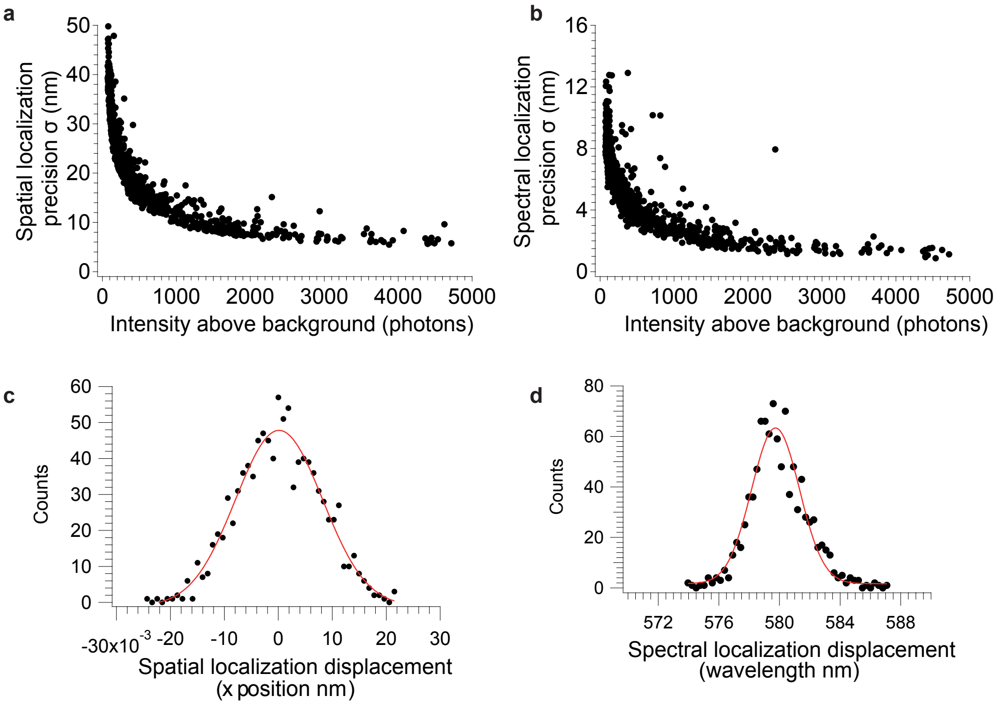

Supplementary Figure 4. Spatial and spectral localization precision of the sPAINT instrument. (**a**) Dependence of the experimentally-obtained spatial localization precision on the measured signal. Each point represents the localization precision and mean integrated fluorescence intensity of a single bead that has been localized ~2000 times, where the asymptote denotes the experimentally determined ultimate spatial localization precision attainable on this particular instrument. (**b**) Corresponding spectral localization precision of the sPAINT instrument. Each point represents the localization precision of the fitted spectrum centre. (**c**) Representative data of the displacement (n = 1000) in the spatial domain from a single bead at ~1500 photons. (**d**) Corresponding representative data of the displacement (n = 996) of the emission peak centre position at 580 nm for the bead in (**c**).

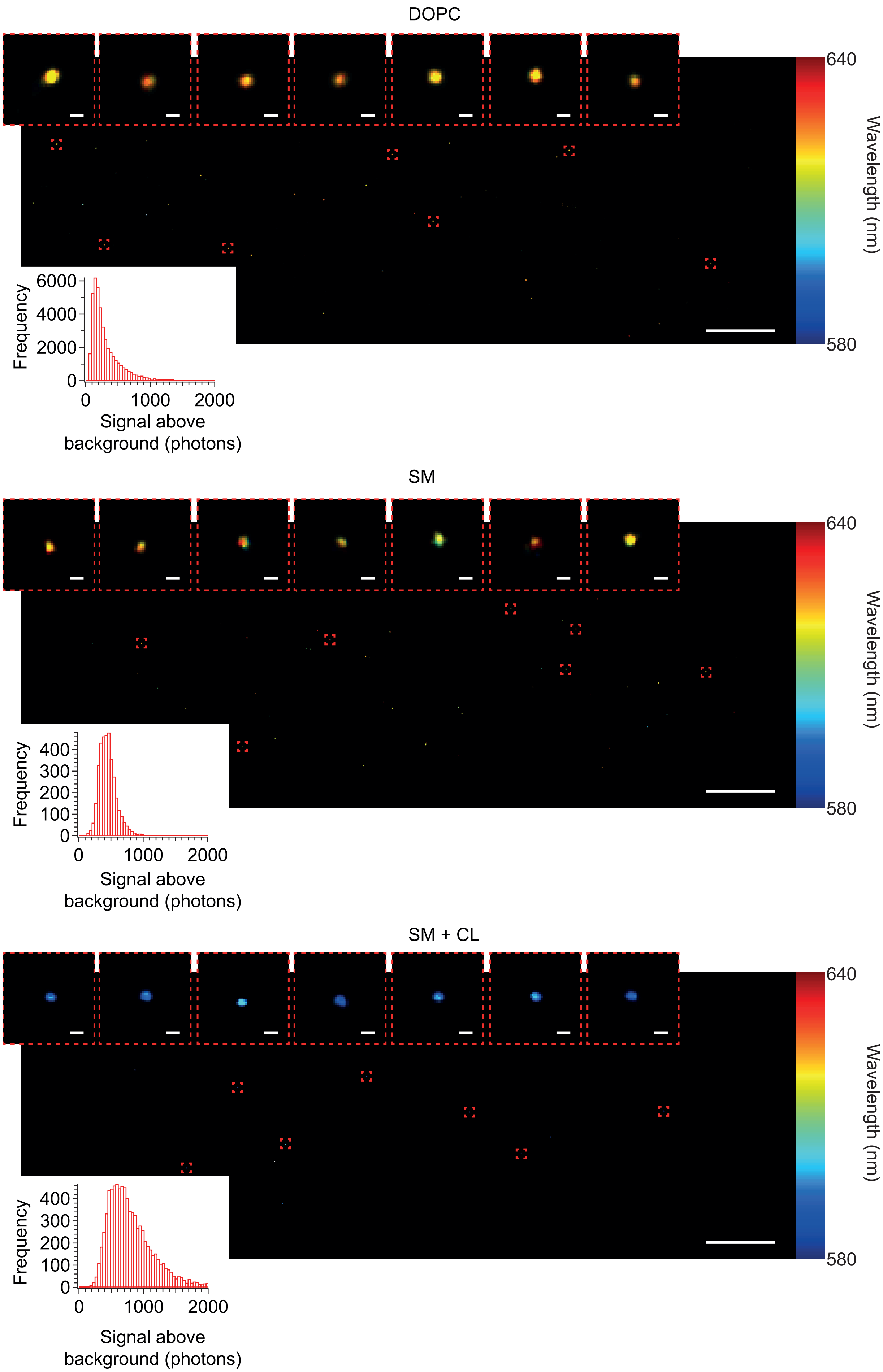

DOPC
640
580
Wavelength (nm)
Frequency
6000
4000
2000
0
0
1000
2000
Signal above background (photons)
SM
640
580
Wavelength (nm)
Frequency
400
300
200
100
0
0
1000
2000
Signal above background (photons)
SM + CL
640
580
Wavelength (nm)
Frequency
400
300
200
100
0
0
1000
2000
Signal above background (photons)

Supplementary Figure 5. Wide field-of-view of LUVs. Representative sPAINT images of LUVs composed of either, DOPC (top), SM (middle), and SM + CL LUVs (bottom). The scale bar is 5 μm (50 nm in the zoom). Histograms of signal intensities for all of the localizations used to generate the sPAINT images are inset into the bottom left of each image.

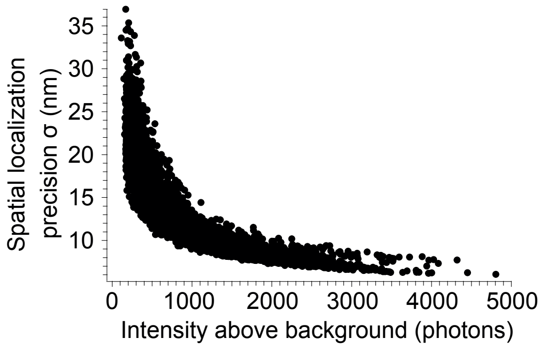

Supplementary Figure 6. Spatial localization precision of the DOPC LUVs. Dependence of the experimentally-obtained spatial localization precision on the measured signal. Each point represents the localization precision and mean integrated fluorescence intensity of LUVs (n is ~ 200) on which NR has been localized ~2000 times, where the asymptote denotes the experimentally determined ultimate spatial localization precision attainable on this particular instrument. The ultimate spatial precision (*i.e.* at infinite photon number) was ~8 nm. Data were obtained from sample DOPC LUVs in various concentrations of the probe dye NR (5 nM – 500 nM).

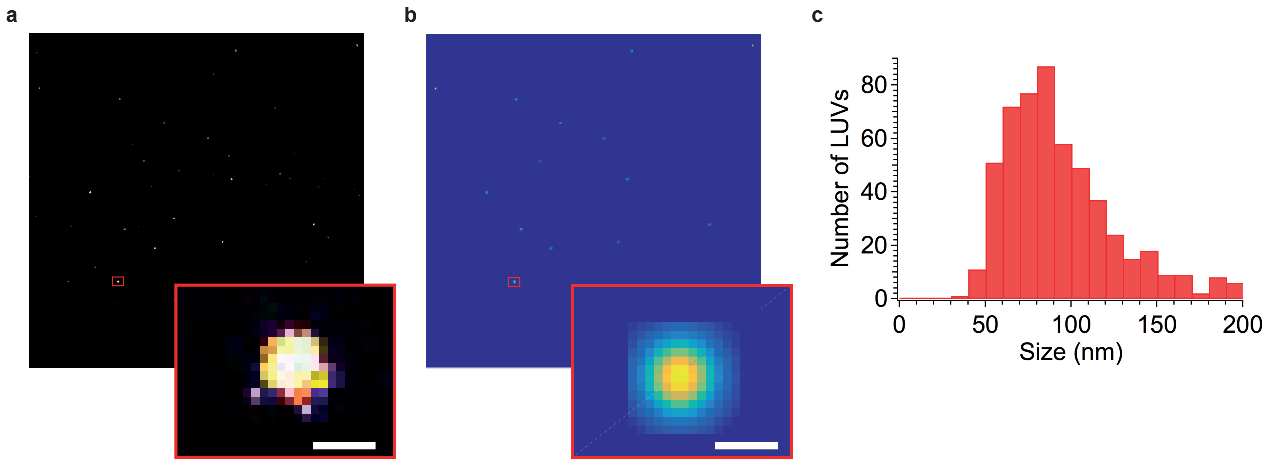

Supplementary Figure 7. Size estimation of LUVs using PAINT.

(**a**) Super-resolution image of ~100 nm unilamellar DOPC vesicles on a glass surface observed during 1000 consecutive frames of ~35 ms per frame.

(**b**) 2D-gaussian representation of LUVs in (a).

(**c**) Histogram of size distribution of LUVs measured by calculated the FWHM of the standard deviation of the Gaussian fit in (b). The median size is 90 nm. Number of LUVs analysed is 592. Dynamic light scattering calculated a diameter of 105 nm. Scale bar 100 nm.

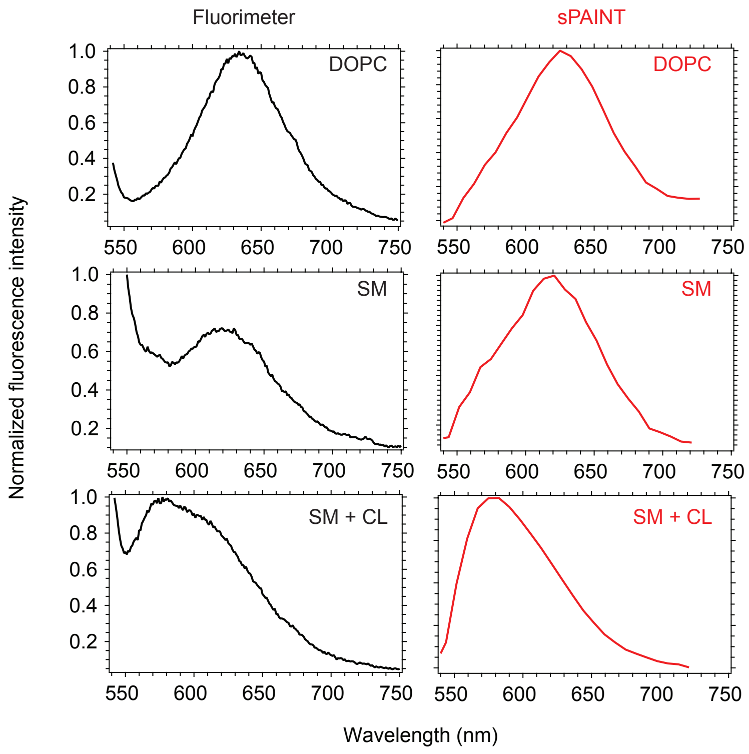

Supplementary Figure 8. NR peak spectral position of LUVs; sPAINT *vs.* bulk. Normalised fluorescence spectra from both a conventional bulk-fluorimeter (black) and ensemble-averaged sPAINT spectra from 1000s of individual spectra (red) for LUVs composed of either: DOPC (top), SM (middle), or SM+CL (bottom).

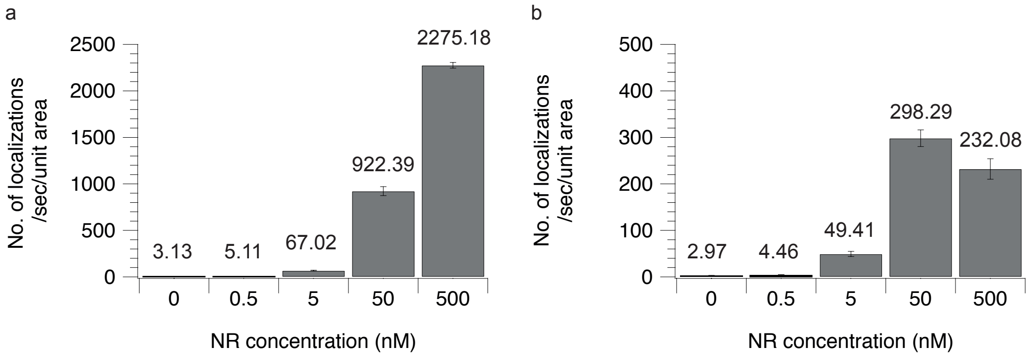

Supplementary Figure 9. Nile red localization rate in LUVs composed of DOPC lipid. (**a**) Total number of localizations per second per field of view (56 µm × 28 µm) as a function of NR concentration. (**b**) Total number of unique $_{\text{localizations}}$ per second per field of view (56 µm × 28 µm) as a function of NR concentration. (**a,b**) Data is the mean ± SEM; *n* = 3. The number of localizations found in (**a**) corresponds to the total number of localizations over all the acquisitions frames - because localization duration can be longer than one single frame, a multidimensional (*x, y, t*) clustering (dbscan) was performed to cluster spatially localised signals appearing on consecutive frames as a single-molecule detection event.

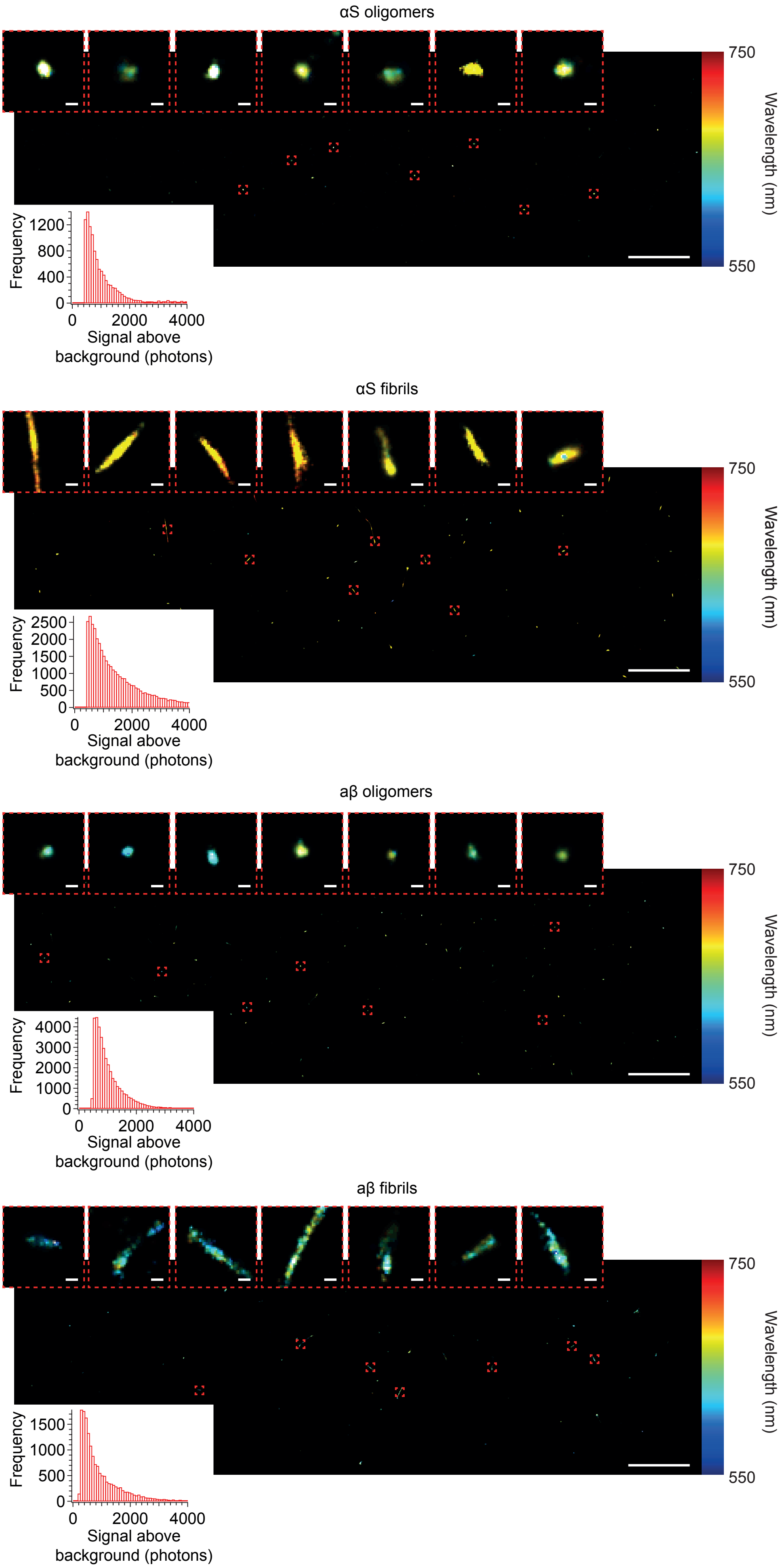

αS oligomers
750
Wavelength (nm)
550
Frequency
1200
800
400
0
0
2000
4000
Signal above background (photons)
αS fibrils
750
Wavelength (nm)
550
Frequency
2500
2000
1500
1000
500
0
0
2000
4000
Signal above background (photons)
aβ oligomers
750
Wavelength (nm)
550
Frequency
4000
3000
2000
1000
0
0
2000
4000
Signal above background (photons)
aβ fibrils
750
Wavelength (nm)
550
Frequency
1500
1000
500
0
0
2000
4000
Signal above background (photons)

Supplementary Figure 10. Representative large field-of-view sPAINT images of protein aggregates. sPAINT images of αS oligomers, αS fibrils, aβ oligomers and aβ fibrils. The scale bar is 5 μm (50 nm in the zoom). Histograms of signal intensities for all of the localizations used to generate the sPAINT images are inset into the bottom left of each image.

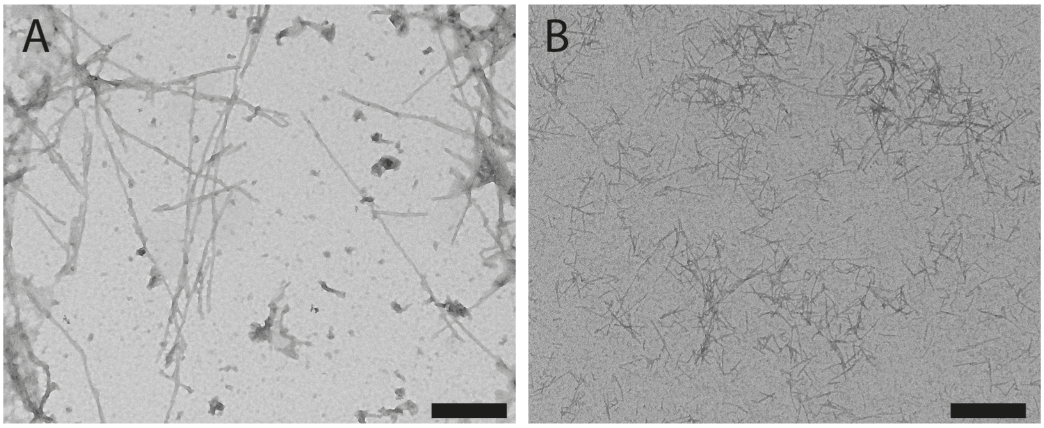

Supplementary Figure 11. Transmission electron micrographs of fibrils. (a) WT αS protein. (b) a$\beta_{1\text{-}42}$ protein. Fibrils were negatively stained with 2 % (w/v) uranyl acetate. The scale bar is 500 nm in length.

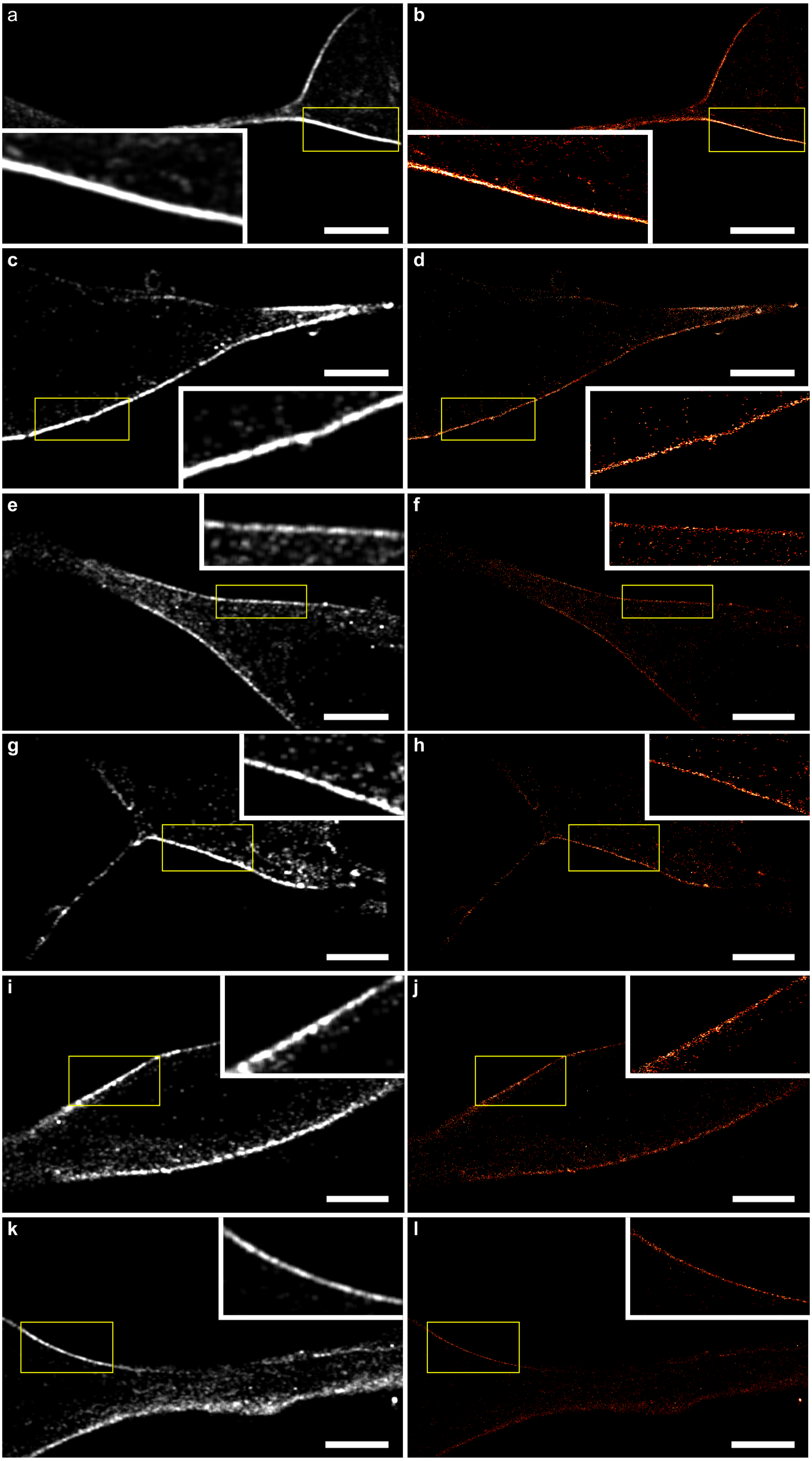
a
b
c
d
e
f
g
h
i
j
k
l

Supplementary Figure 12. Further examples of Diffraction-limited and super resolution images of SHSY5Y cells (fixed and live). Diffraction-limited (**a, c, e, g, i,** and **k**) and super-resolution (**b, d, f, h, j** and **l**) images of SH-SY5Y cell membrane (Scale bar = 5 μm). SH-SY5Y cells were fixed (**a** – **j**) or live (**k** and **l**) and imaged at 20 °C. Super-resolution images were rendered using fitted localizations retrieved typically from 3,000 consecutive frames with 50 ms exposure time.

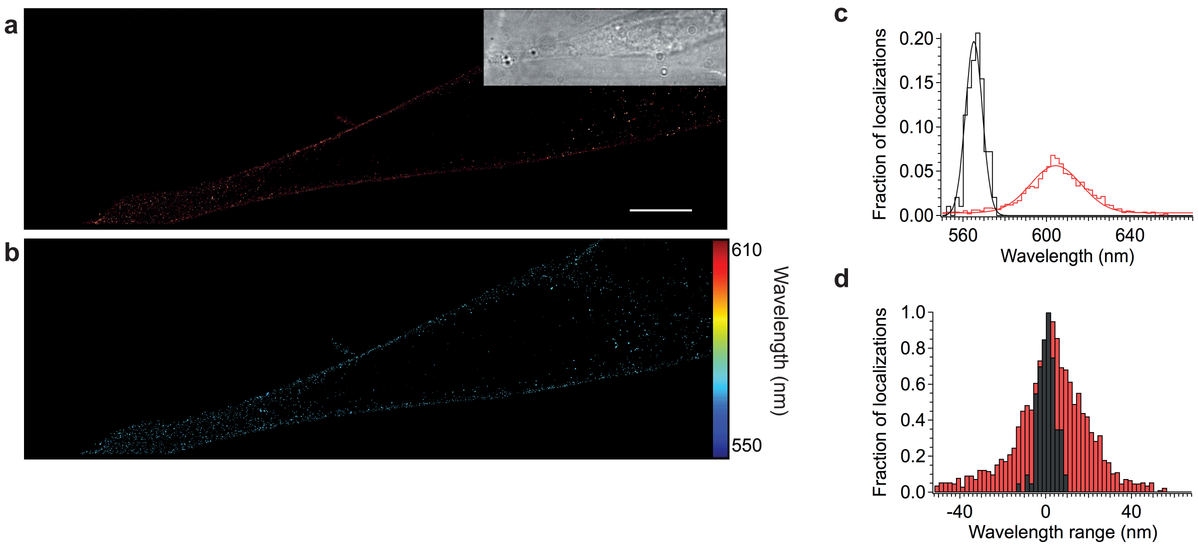

Supplementary Figure 13. sPAINT image of a fixed SH-SY5Y cell using a non-spectrally responsive dye. Super-resolution image of the cell membrane (**a**) and corresponding sPAINT image (**b**) (*n* = 3,886 localizations; scale bar = 5 µm). Note that the lack of spectral-responsiveness manifests itself as little variation in the spectra and therefore a predominantly blue sPAINT image. SH-SY5Y cells were fixed and imaged at 20 °C. Super-resolution images were rendered using fitted localizations retrieved typically from 10,000 consecutive frames with 200 ms exposure time using wheat germ agglutinin (WGA) conjugated to Alexa Fluor 555 (AF555, 0.5 nM). Frequency histogram of fluorescence emission peak (**c**) for WGA-AF555 experiments (black line) or nile red (NR) experiments (red line). The data for the WGA and NR were compiled from consecutive images collected over 90 seconds for comparison. Normalized sPAINT data spread *vs.* wavelength change relative to the peak emission (**d**) (black WGA-AF555, mean 565 nm, SD 5.7 nm; red NR, mean 605 nm, SD 17.2 nm).

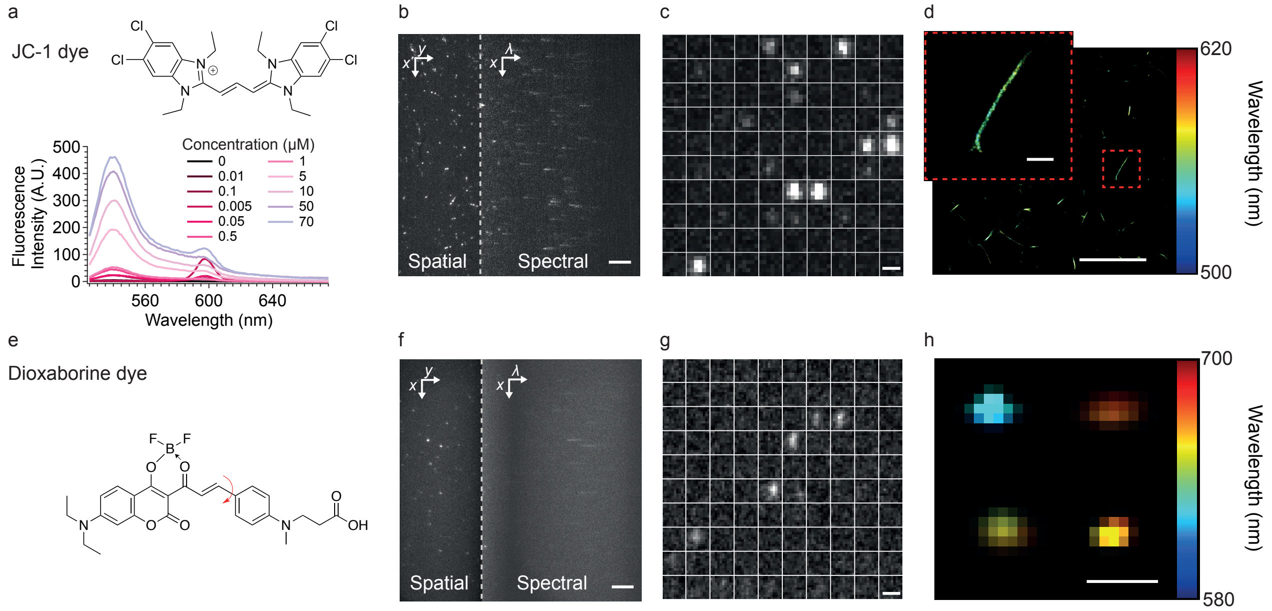

Supplementary Figure 14. md-SR is not limited to hydrophobicity mapping with NR, the principle can map other physical properties using multiple classes of fluorophore. (a) Chemical structure of JC-1 and fluorescence emissions of JC-1 characterized at varying concentrations of αS fibrils with a JC-1 concentration of 0.5 μM at 20 °C using a Cary Eclipse Fluorimeter (Varian, USA). Excitation wavelength was set at 488 ± 10 nm and the fluorescence emission spectrum was collected from 525 nm to 700 nm using a slit width of 5 nm. Photo multiplier was set to 800 V. **(b)** αS fibrils were imaged in 25 nM JC-1 dye by exciting at 532 nm and collecting the emission from 540 nm to 760 nm. 2000 frames were collected with a frame rate of 35 ms. The spatial and spectral components of the image are highlighted (scale bar = 5 μm). **(c)** Montage showing time evolution and fluorescence intermittency of a single JC-1 molecule on an aggregate of αS. Separation between each image is 35ms and scale bar is 500 nm. **(d)** Representative md-SR image of αS fibrils (scale bar = 5 μm and 500 nm in the zoom). **(e)** Chemical structure of the dioxaborine dye. **(f)** Outer Membrane Vesicles (OMVs) purified by tangential flow filtration and ultracentrifugation were immobilized on poly-Lysine coated coverslips. OMVs were imaged in 50 nM dioxaborine dye by exciting at 532 nm and collecting the emission from 567 nm to 647 nm (BLP01-532R Semrock; BP 607/80 AHF). 3,000 frames were collected with a frame rate of 35 ms. The spatial and spectral components of the image are highlighted (scale bar = 5 μm). **(g)** Montage showing time evolution and fluorescence intermittency of single dyes on an individual OMV. Separation between each image is 35ms and scale bar is 500 nm. **(h)** Representative md-SR images of individual OMVs, showing that they dioxoborine has differing wavelengths of emission, corresponding to varying viscosities, which is indicative of their soluble proteins and nucleic acids content heterogeneities (scale bar = 500 nm).

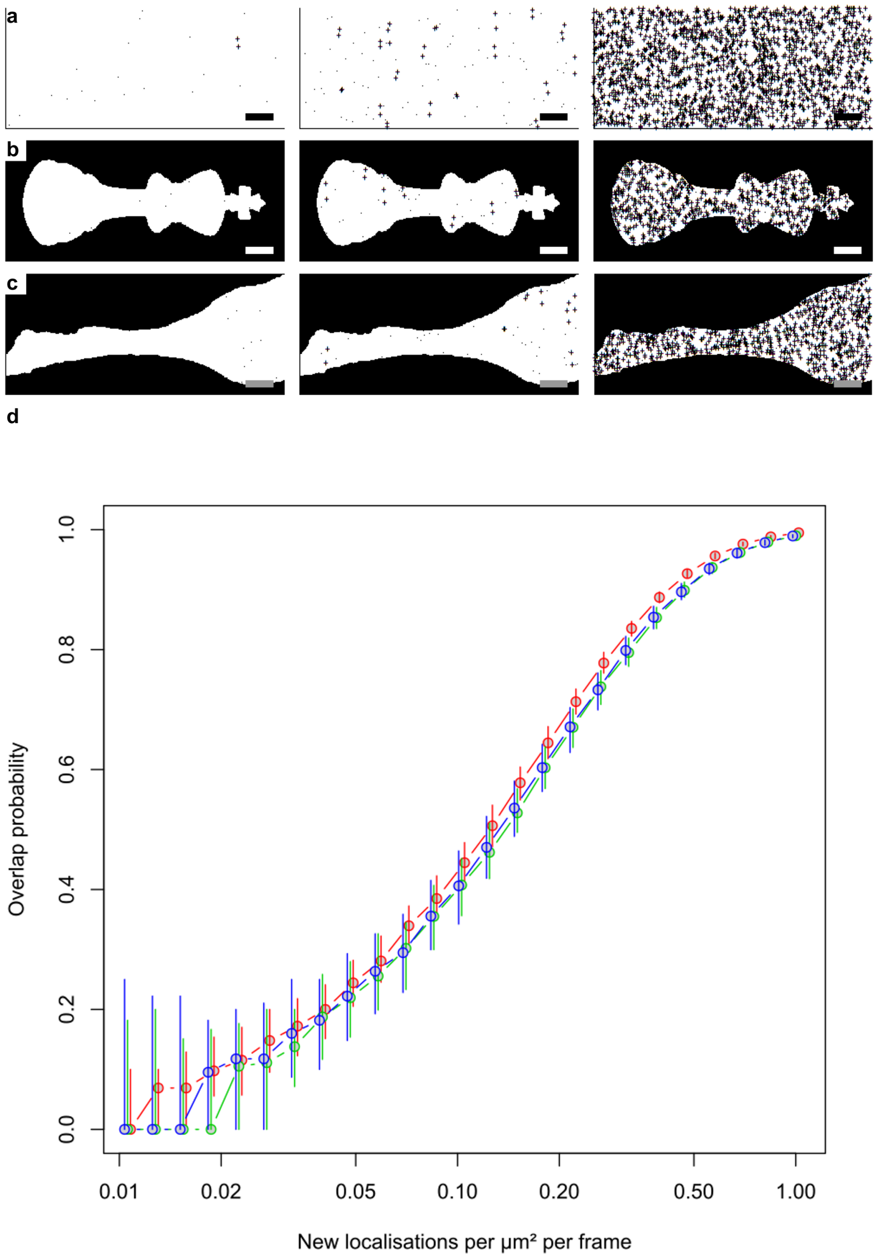

Supplementary Figure 15. Simulations to evaluate the overlapping probability of spectra due to spatial proximity between localisations. (**a, b and c**) Random localisations were generated with densities ranging from low (0.01 localization per µm² per frame) to very high (1 localization per µm² per frame) using different masks shape (**a**-rectangle , **b**- king chess piece or **c**- SH-SY5Y cell shape) . Proximity thresholds of 3 pixels in the x-axis or 30 pixels in the y-axis were used to define if localizations were overlapping (cross) or not (dots). (**d**) Plot of the overlap probability as a function of new localizations per frame. The median (and IQR) probabilities were reported (red curve corresponds to rectangle (**a**), green curve to king chess piece (**b**) and blue curve to SH-SY5Y cell shape (**c**)) as a function of density of new localizations per micrometre squared. On the contrary to localization density, the mask shape seems to poorly modify the overlap probability. It is notable

that typical experimental sPAINT measurements were performed with localization densities ranging from 0.01 to 0.05 where the overlapping probability remains limited. Simulations in **Suppl-ementary Figure 13** were run in R version 3.2.1 (2015). Data were generated in the following manner: For each localization density, 500 frames were generated (pixel size = 110 nm). For each frame Fi, Ni independent and identically distributed random new localizations were generated within a spatial mask using the full area of each shape (rectangle (**a**), king chess piece (**b**) or SH-SY5Y cell (**c**)) assuming a uniform distribution (rpoint function from spatstat package) in which Ni was randomly generated assuming a Poisson distribution. Individual time durations distributed according to a Poisson law of parameter $\lambda = 1.1$ frames were associated to each new localisation. Coordinates of localisations with durations > 1 frame were reported to the Fi+j following frames (j ranging from 1 to max(duration(Ni))). All the generated localizations were then jittered assuming a Gaussian kernel with sd = pointing Precision = 50 nm). The spectral overlapping calculation was evaluated in the following manner: Euclidian matrix distances were computed to calculate |xi - xj| and |yi - yj| (i≠j) distances. Localizations with at least one localization strictly closer than 3 pixels in the x-axis or one strictly closer than 30 pixels in the y-axis were labelled as overlapping. The proportion of overlapping localizations was then calculated for each frame and the median proportion (and IQR) over all frames were reported for each localisation density.

Supplementary Table 1.

Properties of large unilamellar vesicles (LUVs) and NR spectroscopic properties

LUV composition[a]	Phase state[b]	Hydrodynamic radius D_z (nm)[c]	NR fluorescence emission peak (nm)[d]
DOPC	Ld	105	634
SM	Lβ	117	619
SM/CL	Lo	129	587 or 579
PBS buffer	-		659

[a] DOPC is 1,2-Dioleoyl-sn-glycero-3-phosphocholine, SM is sphingomyelin (N-(tricosanoyl)-sphing-4-enine-1-phosphocholine, SM) and CL is cholesterol (SM/CL, 2:1 molar ratio).

[b] Ld, Lo and Lβ correspond to liquid disordered, liquid ordered and solid gel phases for each lipid at 20 °C. For the lipid mixtures, the lipid/ cholesterol ratio was 2:1, mol/mol.

[c] The mean hydrodynamic radius was determined using dynamic light scattering (as described in the materials and methods).

[d] Fluorescence emissions of NR were characterized at saturating concentrations of LUVs with a NR concentration of 0.5 μM at 20 °C using a Cary Eclipse Fluorimeter (Varian, USA). Excitation wavelength was set at 532 ± 10 nm and the fluorescence emission spectrum was collected from 550 nm to 750 nm using a slit width of 5 nm. PM set to 800 V. Background correction from buffer alone was applied. The peak positions measured for NR was highly similar to previous studies.[15]

Supplementary Table 2.

Spectroscopic properties of nile red in protein aggregates

Protein type	Aggregation state	Fluorescence emission peak (nm)[a]
α-synuclein	Fibril	630 ± 1
Aβ	Fibril	626 ± 1
PBS buffer	-	658 ± 2

Fibrils were defined as species that displayed a fibrillar morphology by TEM (**Supplementary Figure 11**).[a] Fluorescence of nile red was recorded at saturating concentrations of protein. Excitation wavelength was 532 nm and the nile red concentration was 0.5 μM. All spectra were recorded at 20 °C using the same fluorimeter and settings as for **Supplementary Table 1.**
