# Supplementary figures and images for: Multi-dimensional super-resolution imaging enables surface hydrophobicity mapping
Source: Nat Commun. 2016 Dec 8;7:13544. doi: 10.1038/ncomms13544 (PMC5155161; doi:10.1038/ncomms13544)

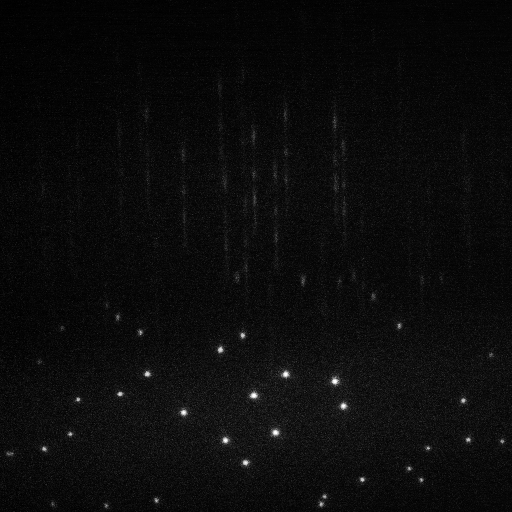

Supplement: Supplementary Data 1 — Spectral calibration example data. TetraSpeckTM beads (0.1 μm, T7297, Invitrogen) were imaged by exciting at 405 nm. The 405 nm excitation beam was directed off a dichroic mirror Di02-R405-25x36 (Semrock, USA) and the emitted fluorescence was filtered using a longpass filter FF02-409/LP-25 (Semrock, USA). 100 frames were collected with a frame rate of 35 ms. [file ncomms13544-s3.zip › STEP1_Calibration_405_Ex.tif]
