# Supplementary material for: Multi-dimensional super-resolution imaging enables surface hydrophobicity mapping
Source: Nat Commun. 2016 Dec 8;7:13544. doi: 10.1038/ncomms13544 (PMC5155161; doi:10.1038/ncomms13544)
Supplement: Supplementary Data 2 — Analysis Code 1 of 4. ImageJ plugin used to determine the maxima in the spectra in the sPAINT images from the SR localizations in the calibration file. [file ncomms13544-s4.html]

macro sPAINTcalib{
// JuG le 09/03/2016
//macro for sPAINT wavelength calibration using tetraspeck Beads
name = getTitle;
print(name);
dir = getInfo("image.directory");
//print(dir);
if(nSlices()<2){
exit("Stack required");
}
delta = newArray(3);
wvlEx = newArray(3);
Dialog.create("sPAINT");
Dialog.addNumber("Distance to 512 peak (px):", 192);
Dialog.addNumber("Green Wavelength (nm):", 512.7);
Dialog.addNumber("Distance to 581 peak (px):", 220);
Dialog.addNumber("Orange Wavelength (nm):", 581.5);
Dialog.addNumber("Distance to 676 peak (px):", 260);
Dialog.addNumber("Red Wavelength (nm):", 676.5);
html = ""
+"

## Help

"
+"Distance Z0-Z1 is the distance   
"
+"btw the spatial and spectral spots (in px)  
"
+"";
Dialog.addHelp(html);
Dialog.show();
delta[0] = Dialog.getNumber();
wvlEx[0] = Dialog.getNumber();
delta[1] = Dialog.getNumber();
wvlEx[1] = Dialog.getNumber();
delta[2] = Dialog.getNumber();
wvlEx[2] = Dialog.getNumber();
run("Set Scale...", "distance=0");
//ouvrir le fichier de localisation
if (isOpen("Results")) {
selectWindow("Results");
run("Close");
}
file = 0;
list = getFileList(dir);
for (i=0; i 0;
bool2 = centre[k] < 30 && centre[k] > 0;
bool3 = width[k] < 20 && width[k] > 1.5;
bool4 = distcentre[k] < (delta[j] + 20) && distcentre[k] > (delta[j] - 20);
use[k] = bool1 & bool2 & bool3 & bool4;
}
for (i=0; i
