# Supplementary material for: Multi-dimensional super-resolution imaging enables surface hydrophobicity mapping
Source: Nat Commun. 2016 Dec 8;7:13544. doi: 10.1038/ncomms13544 (PMC5155161; doi:10.1038/ncomms13544)
Supplement: Supplementary Data 4 — Analysis Code 3 of 4. ImageJ plugin used to determine the maxima in the spectra in the sPAINT images from the SR localizations in experimental sPAINT data. [file ncomms13544-s6.html]

macro sPAINT{
// JuG le 09/03/2016
name = getTitle;
print(name);
dir = getInfo("image.directory");
//print(dir);
if(nSlices()<2){
exit("Stack required");
}
Dialog.create("sPAINT");
Dialog.addNumber("Distance Z0-Z1:", 250);
Dialog.addNumber("Beta 0: intercept", 0);
Dialog.addNumber("Beta 1: lambda", 0);
Dialog.addNumber("Beta 2: X:",0);
Dialog.addNumber("Beta 3: Y:", 0);
html = ""
+"

## Help

"
+"Title can be left empty   
"
+"Distance Z0-Z1 is the distance   
"
+"btw the spatial and spectral spots (in px)  
"
+"";
Dialog.addHelp(html);
Dialog.show();
delta = Dialog.getNumber();
b0 = Dialog.getNumber();
b1 = Dialog.getNumber();
b2 = Dialog.getNumber();
b3 = Dialog.getNumber();
run("Set Scale...", "distance=0");
//ouvrir le fichier de localisation
if (isOpen("Results")) {
selectWindow("Results");
run("Close");
}
file = 0;
list = getFileList(dir);
for (i=0; i 0;
bool2 = centre[k] < 30 && centre[k] > 0;
bool3 = width[k] < 20 && width[k] > 1.5;
bool4 = distcentre[k] < (delta + 20) && distcentre[k] > (delta - 20);
use[k] = bool1 & bool2 & bool3 & bool4;
print("ampli: " +ampli[k]+"\ncentre: "+centre[k]+"\nwidth: "+width[k]+"\ndist: "+distcentre[k]);
print("dist: "+distcentre[k]);
print("use :"+use[k]);
}
print("\\Clear");
print("X,Y,X.sd,Y.sd,Background,Noise,Signal,Frame,Lambda,Use,distZ0Z1");
for (i=0; i
